# Dancing for Taste: The Impact of AI and Human Influencers on Aesthetic Perception and Purchase Intention for Food Products

**DOI:** 10.3390/foods15050928

**Published:** 2026-03-06

**Authors:** Defang Sha, Kai Zhang, Yanlong Wei

**Affiliations:** 1 Zhujiang College, South China of Agricultural University, Guangzhou 510900, China; shadefang@scauzj.edu.cn; 2 Faculty of Fine and Applied Arts, Khon Kaen University, Khon Kaen 40002, Thailand; 3 Department of Mechatronics Engineering, Harbin Institute of Technology, Harbin 150030, China

**Keywords:** aesthetic perception, AI influencer, dance style, sensory marketing, food product, influencer humanization

## Abstract

As digital food marketing increasingly relies on short-form visual content, understanding how performance aesthetics interact with influencer characteristics becomes critical. The present research examines the effects of the combination of the influencer type (AI or human) and the dance style (modern or traditional) on the attitudes of consumers towards food products. We show that aesthetic congruence, where human and traditional dance performers are matched with AI and modern dance performers, respectively, can result in increased purchase intention in two preregistered experiments (*N* = 462). The interaction effect was significant in Study 1, and this emphasises the strength of congruent combinations. Study 2 also indicated that these influences are mediated through two different aesthetic feelings: cultural aesthetics and technological aesthetics. Also, one of the real measures of behaviour was that aesthetic congruity affected the real purchase actions. These results indicate that performed aesthetics could be used to improve the effectiveness of digital food marketing, provided it is strategically aligned with influencer identity.

## 1. Introduction

With the era of immersive digital experiences, the marketing of food is not only associated with taste and nutritional values [1,2,3]. It is now becoming more sensory, especially visually appealing, to influence the perception and actions of consumers [4,5,6]. Influencer marketing and short-form video services have become mainstream channels of food and food product advertising in such a setting [7,8]. These platforms tend to depend on performance-driven content (songs and dances) [9,10] to produce emotionally stimulating stories that appeal to their desired audience.

Consequently, the emergence of artificial intelligence (AI) influencers, digital characters that resemble human behaviour and expression, has presented novel perspectives on the relationships between consumers and brands [10]. Although AI influencers are controllable and novel, it is unclear whether they are less authentic, trustworthy, and persuasive than human influencers [11,12,13]. Dance, being a visually intense and emotionally loaded form of communication [14,15,16], offers an unusual perspective to examine these differences. However, there are only a handful of studies that have explored the ways in which various types of influencers (AI or human) and various types of dance styles (modern or traditional) can influence consumers’ sensory perceptions and behaviour outcomes, particularly when it comes to agricultural produce.

Using the theories of aesthetic psychology [17,18] and sensory marketing [19,20], the current study explores the way particular influencer-dance combinations stimulate various types of aesthetic experience, cultural aesthetics (connected to familiarity, tradition, and nature), and technological aesthetics (related to novelty, precision, and digital expression) [21,22,23,24]. We claim that these aesthetic responses act as mediators whereby digital performances are able to impact purchase intentions regarding agrifood products.

These questions will be addressed through two experiments. In Study 1, the major effects of influencer type and dance style are explored in terms of the purchase intention of consumers. Study 2 is an examination of a dual-mediation model whereby the relationships between influencer-dance combinations and consumer purchase intention are mediated by cultural aesthetics and technological aesthetics. Taken together, these studies add to the body of knowledge by showing that the aesthetic form of digital content, which is influenced by the performer and the performance, has a decisive contribution to consumer behaviour related to food.

## 2. Theoretical Background and Hypotheses Development

### 2.1. Sensory Marketing and Aesthetic Perception in Food Promotion

The concept of sensory marketing has been well received in the recent past as an effective model of changing the behaviours of customers via multisensory stimulation [25,26]. In contrast to conventional methods that mostly deal with informational appeals, sensory marketing revolves around the manner in which people see, hear, taste, touch, and smell products (the five senses) [9]. In food and agriculture-related products, in which taste is a major motivator, visual and auditory cues are playing a growing role in determining pre-consumption judgement [5,27].

In this list of senses, visual perception is the most common modality of food advertising in the digital world. Researchers have shown that visual characteristics like colour, shape, motion, and symmetry may play a significant role in influencing perceptions of freshness, healthiness, and quality [5,6,28]. Dance, choreography, and influencer appearance are important visual triggers in the sphere of short-form video marketing, which appeals to the consumer both emotionally and cognitively [8,29]. These visual signals not only trigger aesthetic delight but also act as symbolic conveyors of meaning, particularly when combined with cultural or technological concepts.

It should also be noted that the concept of aesthetic perception in sensory marketing is not monolithic. As per the concept of aesthetic psychology [17,30], people react to stimuli differently depending on the dimensions that include familiarity, complexity, novelty, and cultural resonance [31]. The recent sources highlight the fact that aesthetic experiences may be classified, including cultural aesthetics, which are based on tradition, nature, and emotional warmth [21,22,32], and technological aesthetics, which are related to novelty, digital sleekness, and visual precision [23,24,33]. These differences have a particular relevance in the case of online food promotion, where not only the form of performance (e.g., dance styles) but also the identity of the performer (e.g., AI or human) influences the evaluation of the product by customers.

Therefore, the current study sees aesthetic perception as an inherent psychological process that plays a central role in connecting digital performance content with behaviour related to food. By making these distinctions between cultural and technological aesthetic experience, we seek to uncover the manner in which various sensory tactics may generate divergent emotions and thoughts and eventually affect the purchase intention of consumers in the food products domain.

### 2.2. Influencer Type: AI vs. Human in Digital Food Marketing

The spread of social media influencers has transformed the way people interact with food-related information and make consumption choices. Historically, there were human influencers, frequently food bloggers, nutritionists, or lifestyle creators [34], who dominated the influence on the perception of food quality, taste, and authenticity among people. Human influencers can evoke feelings of emotional attachment and trust using storytelling, personal endorsement, and cultural resonance, especially when they promote agricultural or natural products that have deep connections to the concepts of locality, sustainability, and tradition [35,36].

Nevertheless, the fast development of AI technologies brought about a new age of virtual influencers, who are digital avatars that simulate the human look, act, and communicate [12,37]. Such AI-driven influencers may be completely algorithmic or partially human-controlled, providing exact branding fit, scalability, and futuristic charm [10]. Regardless of their artificiality, AI influencers are becoming popular in various industries, such as the beauty industry, fashion industry, and lately the food and lifestyle industry.

Nevertheless, studies indicate that consumer responses to AI influencers are uncertain. On the one hand, AI influencers may elicit a feeling of curiosity and interest because of their newness and fluency of design [11]. On the other hand, they are often not seen as authentic or relationally warm like human influencers, which are important aspects of low-involvement and emotionally driven contexts like food marketing [38]. It is especially prominent when it comes to agricultural goods, since the sense of trust in the origin, nature, and emotion plays a central role in the decision of the consumers about purchasing them [39]. Regarding aesthetic reaction, human influencers are more likely to elicit feelings of warmth, familiarity, and cultural connectedness, particularly when performing in traditional food-related events [34]. On the other hand, AI influencers can evoke technological aesthetics, which are newness, accuracy, and a sense of futurism [24]. Therefore, the kind of influencer could influence not just the perceived credibility of food products but also the aesthetic filter that is used to assess them, particularly on visually oriented social media platforms such as TikTok or Instagram Reels.

With the development of technology, the way influencers work and the methods of communication are constantly evolving. Intelligent technologies (such as AI speech synthesis and virtual image generation) have provided broadcasters with new tools and platforms, but they also require influencers to have the ability to adapt to new technologies and achieve human–machine collaboration. For example, AI influencers can handle large amounts of data and repetitive tasks, while human influencers focus on creativity, emotions, and the presentation of in-depth content. As early as 2016, journalists and AI technologies took the first step toward integration. The development trends in media technology have become clearer, showing the characteristics of an era where “everything is media, human–machine symbiosis, and self-evolution.” The AI virtual influencer technology is a departure from the past, offering advantages such as “strong professionalism, cost-effectiveness, and broad application scenarios.” Human hosts no longer compete on “voice” but on “AI tool proficiency + creativity,” treating AI as a “partner” rather than a “substitute.”

Based on such dynamics, the research explores what role the type of influencer (AI or human) plays in relation to the dance style in affecting the perception of aesthetics by consumers and subsequent purchasing intent related to food products.

### 2.3. Dance Style as a Visual Sensory Cue

Movement-based visual cues, including gestures, body language, and dance, are important in the field of sensory marketing to influence the emotional and cognitive perceptions of products [27]. In particular, dance is a form of visual and rhythmic richness that expresses emotion, story, and symbolism. It has become more popular in short-form video material, particularly on apps such as TikTok and Instagram reels, where it is used as a way to engage users and create an emotional connection [29]. Dance may be seen as a non-verbal sensory cue to use on food and agrifood products to make the viewer have a better aesthetic experience, as well as emotional participation with the advertised product.

It is important to note that dance is not an undifferentiated concept but involves various forms with different emotional and aesthetic effects. The traditional or cultural dances (e.g., ethnic choreography and folk dances) are commonly viewed as warm, familiar, and natural, and they evoke nostalgia, authenticity, and cultural pride [40,41]. These dances can be particularly resonant with the values associated with food products, naturalness, locality, and attachment to the land. On the other hand, the contemporary dance practices (e.g., hip-hop and electronic choreography) tend to focus on accuracy, innovation, and computerization that might be more compatible with technological aesthetics and futuristic marketing [42].

The interaction between dance style and influencer type is also a factor that complicates things. If a human influencer performs a traditional dance, the duo can serve to reinforce the idea of cultural embeddedness and emotional sincerity, which are both known to be factors that increase trust and purchase intention in food marketing [43]. Conversely, an AI influencer dancing to a modern dance can contribute to the impression of digital sophistication and novelty, which will activate a different yet equally influential persuasive path, particularly among the customers who are technologically advanced or innovative-minded [44].

Under these circumstances, we suggest that the efficacy of dance style in advertising agricultural goods is determined by the compatibility between the dance style and the type of influencer. In particular, our hypothesis is that human-traditional combinations would evoke stronger purchase intent through cultural harmonisation, whereas AI-modern combinations would through technology-based charm. Based on this, we offer the following hypotheses:

**H1:**  *There is an interaction effect between the influencer type and the dance style on consumers’ purchase intentions for food products.*

**H1a:**  *Human (vs. AI) influencers performing traditional dances will elicit higher levels of purchase intentions.*

**H1b:**  *AI (vs. human) influencers performing modern dances will elicit higher levels of purchase intentions.*

### 2.4. Aesthetic Experience Typology: Cultural vs. Technological Aesthetics

Aesthetic perception has been considered to be one of the key factors in consumer behaviour, particularly when it comes to areas in which sensory involvement and emotional interpretation play an important role [4,45]. When sensory marketing is used, aesthetic responses are not just concerned with beauty or visual attractiveness but are part of meaning-making processes that shape what people think about products, brands, and the situation in which they are displayed. The scholarship has pointed out that aesthetic experience can be categorised into two experiential trends: cultural aesthetics and technological aesthetics [21,22,23,24,32,33]. These are not merely visual preferences but also involve more profound schemas in terms of authenticity, innovation, tradition, and digital fluency that consumers have in mind.

Cultural aesthetics refers to values associated with tradition, emotional warmth, nature, and familiarity. These experiences are typically a result of imagery that evokes memories of village life, artisanal craftsmanship, or culturally rooted acts. In the context of food and agriculture, cultural aesthetics symbolise authenticity, natural origin, and emotional connection, which enhance the level of trust and emotional attachment to a product [21,22,32]. When human influencers perform traditional dances, these signals are amplified, enhancing the sense of authenticity and emotional significance of the product, which is crucial in agricultural food marketing.

On the other hand, technological aesthetics are characterised by novelty, precision, smoothness, and digitalization. These aesthetics arise from highly stylized, modern, or futuristic visual content, particularly when created by AI or augmented through digital devices [23,24,33]. Modern dance routines performed by AI influencers typically imply the concepts of technological control, efficiency, and modernity, evoking coolness and innovation-driven assessments, even for products traditionally associated with nature, such as food. Thus, technological aesthetics emphasise the novelty and futuristic appeal of the product and influencer.

To further clarify the distinctions between these two aesthetic types, we present a conceptual diagram contrasting cultural aesthetics and technological aesthetics with alternative explanations, such as trust, authenticity, novelty, and modernity. This diagram will help delineate how these aesthetics relate to other psychological constructs and provide a clearer theoretical grounding for the present study.

Based on these distinctions, we hypothesise that cultural aesthetics and technological aesthetics operate as parallel mediators, affecting the influence of influencer type and dance style on consumer purchase intentions. Specifically, cultural aesthetics mediate the influence of human influencers performing traditional dances, while technological aesthetics mediate the influence of AI influencers performing modern dances.

**H2:**  *The indirect effect of influencer type on purchase intention through cultural aesthetics is moderated by dance style, such that this effect is positive and significant for traditional dances (when the influencer is human vs. AI), but not for modern dances.*

**H3:**  *The indirect effect of influencer type on purchase intention through technological aesthetics is moderated by dance style, such that this effect is positive and significant for modern dances (when the influencer is AI vs. human), but not for traditional dances.*

**Figure 1:**
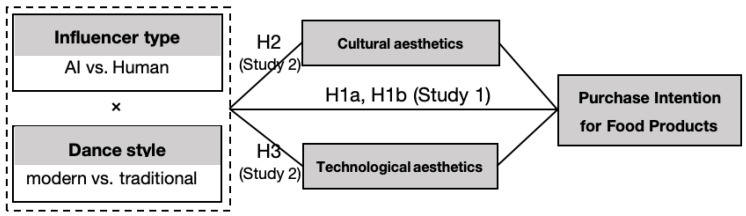
Research model.

These hypotheses are grounded in psychological theories, such as schema congruity theory [17] and processing fluency [27], which suggest that consumers’ aesthetic responses are influenced by the compatibility between the performer’s identity (human vs. AI) and the performance style (traditional vs. modern). Specifically, the alignment between influencer type and dance style enhances aesthetic experience and, in turn, influences purchase intentions. Additionally, the human–computer interaction (HCI) framework [11] provides further support for understanding how AI influencers can evoke technological aesthetics through their futuristic and digital performances. The research model is illustrated in Figure 1.

## 3. Study 1: Main Effects of Influencer Type and Dance Style on Purchase Intention

The research is aimed at revealing the significant effect and the interaction between the type of influencer (AI or human) and the style of dance (modern or traditional) on the purchasing intentions of food products by consumers. Extending existing studies in the field of sensory marketing and visual persuasion, Study 1 examines H1, H1a, and H1b, which are based on the influence of visual performance combinations on consumer behavioural reactions.

### 3.1. Method

The recruitment was done using a professional Chinese online research platform, Credamo, which registered 250 participants in total [46]. Each participant was paid 30 RMB (about 4.20 USD) to take part in the survey. The remaining 221 valid samples were kept in the analysis after eliminating 19 inattentive or incomplete responses (*M_age_* = 28.9, *SD* = 8.91, 55.7% female). Inattention was measured using two methods: (1) attention checks, where participants were asked to select a specific response to a dummy question (e.g., “Please select ‘Strongly Agree’ for this question”) and (2) response time analysis, where responses with a total time of less than 3 min were excluded, as they were deemed too fast to reflect thoughtful engagement.

To calculate the necessary sample size, we applied the G*Power software (version 3.1.9.7.) [47]. In the case of the 2 × 2 between-participants design that was used in the present experiment, the minimum number of participants needed to detect a medium effect size (f = 0.25) at a statistical power of 0.85 and an alpha level of 0.05 was N = 38. Our total sample size (N = 221) was therefore larger than the minimum requirement, which will provide sufficient statistical power to test hypotheses.

The participants were randomly allocated into one of the four experimental conditions of the 2 (influencer type: human vs. AI) × 2 (dance style: traditional vs. modern) between-subjects design. All participants viewed a short-form video of approximately 60 s that advertised a local food product (Hami melon). All videos were standardised in terms of camera angle, background setting, lighting, and video length to achieve experimental control [48]. All the conditions had the same background music, which was the same instrumental track but modified to a different tempo to suit the rhythm of the dance. All videos featured a visual representation of the Hami melon without any spoken narration to eliminate verbal bias. Upon watching the video, participants filled out a questionnaire that assessed purchase intention, manipulation checks, and demographics. Prior to data collection, the design, hypotheses, sample size, and analysis plan of this research were preregistered on the AsPredicted website (https://aspredicted.org/56q4uj.pdf) (accessed on 9 December 2025) [49].

### 3.2. Measures

Buy Intention. It was assessed through a 3-item scale based on Dodds et al. (1991) [50] and was scored on a 7-point Likert scale (1 = strongly disagree, 7 = strongly agree). The items were: I would consider purchasing the product; I will probably buy this product soon; This product is attractive to such an extent as to affect my buying process. The reliability of the scale was high (*α* = 0.83).

Manipulation checks. Two items were used to measure perceived influencer type (the influencer appeared to be a real human) and dance style (the dance felt modern), measured with 7-point semantic differential scales.

### 3.3. Results

In order to examine the proposed influence of both influencer type and dance style on purchase intention, a 2 × 2 between-subjects ANOVA (2 levels of influencer: human and AI; 2 levels of dance: traditional and modern) was used in SPSS 27.0. The dependent variable was the participants’ intention to buy the Hami melon product after watching the video.

Manipulation Checks. An independent-samples t-test revealed a significant difference between conditions: participants in the AI condition reported significantly lower scores (*M_AI_* = 4.06, *SD_AI_* = 0.86) than did those in the human condition (*M_human_* = 4.94, *SD_human_* = 0.79, *t*(219) = −7.92, *p* < 0.001), confirming successful manipulation of the influencer type. For dance style, participants assigned to the modern-style condition (*M_modern-style_* = 5.02, *SD_modern-style_* = 0.84) reported significantly more agreement than those in the traditional-style condition (*M_traditional-style_* = 3.99, *SD_traditional-style_* = 0.85, *t*(219) = −9.02, *p* < 0.001). These results validate the success of both manipulations.

Main Effects. The main effect of influencer type on purchase intention was not statistically significant (F(1, 217) = 0.01, *p* = 0.92). Similarly, the main effect of dance style was also non-significant (F(1, 217) = 0.07, *p* = 0.80). These findings suggest that neither influencer type nor dance style alone was sufficient to significantly shift participants’ purchase intentions.

Interaction Effects. Importantly, a significant interaction effect was observed between influencer type and dance style (F(1, 217) = 35.11, *p* < 0.001, partial *η*^2^ = 0.13), providing support for H1. This result indicates that the effectiveness of the influencer in promoting food products depends on the style of dance they perform.

**Figure 2:**
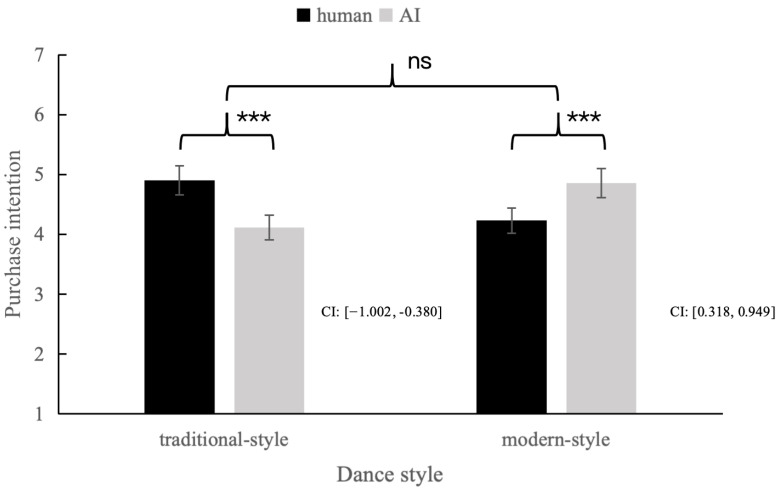
Interaction effects of the influencer type and dance style on adoption intention. The error bars show the standard errors of the means. Note: that *** denotes *p* < 0.001, ns denotes not significant.

Simple Effects Analysis. Follow-up tests revealed (Figure 2) that in the traditional-style dance condition, participants exposed to human influencers reported significantly higher purchase intentions (*M_human_* = 4.90, *SD_human_* = 0.82) than those who viewed AI influencers (*M_AI_* = 4.11, *SD_AI_* = 0.80, *p* < 0.001). Conversely, in the modern-style dance condition, participants exposed to AI influencers showed significantly higher purchase intentions (*M_AI_* = 4.86, *SD_AI_* = 0.79) than those exposed to human influencers (*M_human_* = 4.23, *SD_human_* = 0.89, *p* < 0.001). These findings support H1a and H1b, showing that purchase intentions were highest when the influencer–dance pairing was congruent: human with traditional dance and AI with modern dance.

### 3.4. Discussion

The first study offers initial empirical evidence for the hypothesis H1, stating that influencer type and dance style can be used to influence consumer behaviour. Although none of the variables had a significant main effect alone, they together created a meaningful impact on the purchase intentions of the customers towards a food product. The results are consistent with the aesthetic congruence hypothesis: the presentation is more convincing when the symbolic significance of the dance style is compatible with the perceived identity of the influencer [51]. People who are human influencers dancing in the traditional style can promote a greater sense of cultural authenticity and trust, particularly when it comes to such products as Hami melon. Conversely, AI influencers combined with contemporary dance may attract people looking to try something new and innovative online, including food. The given interaction pattern is the prelude to Study 2, which investigates the mechanisms behind such effects, namely, aesthetic perceptions (cultural or technological) that mediate these effects.

## 4. Study 2: Aesthetic Mediation Model

In order to elaborate on the processes behind the interaction effect found in Study 1, Study 2 examined whether aesthetic perception (cultural or technological aesthetics) can be a mediator between the effects of influencer type and dance style on purchase intention. Additionally, it was a real behavioural choice component that was included in this study, which improves the external validity of the results.

### 4.1. Method

Study 2 was conducted offline at South China Agricultural University, Zhujiang College. A total of 248 students voluntarily participated in exchange for a 10 RMB compensation. Following the exclusion of 18 invalid responses, 230 valid participants were included in the analysis (*M_age_* = 21.9, *SD* = 3.11, 47.8% female).

To achieve sufficient statistical power, we estimated the necessary sample size using the G*Power software [47]. In the present study, which used a 2 × 2 between-participants design, the minimum number of participants needed to identify a medium effect size (f = 0.25) with a statistical power of 0.85 and an alpha level of 0.05 is N = 38. We ended up with a total of 230 participants, which is much higher than this threshold, which means that our statistical adequacy is highly robust.

The experimental design was the same as Study 1: a 2 (influencer: human or AI) × 2 (dance style: traditional or modern) between-subjects design. Every participant viewed a brief promotional video (around 60 s) in a controlled classroom setting [48]. The product used in this research paper is a fresh mandarin orange, which is an example of a typical seasonal food product.

To be more realistic, it was stated to the participants that the fruit used in the video is local and can be obtained on-site following the experiment. Upon viewing the video, they filled out the same questions (questionnaire) that had been used in Study 1. At the conclusion of the session, participants were informed that they could use the 10 RMB reward to buy one box of the mandarin that was tested in the experiment. Their choice (purchase or no purchase) was registered as a behavioural outcome. The design, hypotheses, sample size, and analysis plan of the present study were registered on the AsPredicted platform before data collection (https://aspredicted.org/na2rh4.pdf) (accessed on 9 December 2025) [49].

### 4.2. Measures

Purchase Intention. Same as Study 1, measured with 3 Likert-scale items (*α* = 0.85).

Perception of Aesthetics. Two independent constructs were evaluated to determine the perceived aesthetic experience. Cultural Aesthetics—assessed with a 4-item scale (e.g., “This video was culturally rich”; “The performance is linked to tradition”), which is an adaptation of Ye et al. (2024) and Coleman (2005) [21,22] (α = 0.91). Technological Aesthetics—assessed with a 4-item scale (e.g., “The video appeared modern and tech-savvy”; “It felt digitised”), which is derived after Schummer et al. (2009) and Montani (2020) [24,33] (α = 0.94). Each of the items had a 7-point Likert scale (i.e., 1 = strongly disagree, 7 = strongly agree). Pretests established the discriminant validity of both scales.

Alternative Mediators. In order to consider other possible explanations, a number of psychological variables that are frequently investigated were added as alternative mediators. A scale with three items, which was based on the work of Levin et al. (2006), was used to assess Perceived Trustworthiness (i.e., the influencer is perceived to be trustworthy and reliable, = 0.89) [52]. A 3-item scale (i.e., the influencer appears real and genuine, = 0.88), which was adapted based on the study of Cinelli et al. (2020) [53], was used to measure Perceived Authenticity. Attitude Toward the Influencer/Product was measured with a 3-item semantic differential scale (i.e., unfavourable–favourable, unpleasant–pleasant, = 0.91) according to Du et al. (2023) [54]. The ratings of all items were done on 7-point Likert scales (1 = strongly disagree, 7 = strongly agree).

Behavioural Choice. The choice to spend 10 RMB to buy a box of mandarins was coded as a behavioural proxy of purchase behaviour (1 = purchased; 0 = did not purchase).

Manipulation checks. Two items were used to determine the perceived influencer type (“The influencer appeared to be a real human”) and dance style (“The dance felt modern”), with participants responding to it using 7-point semantic differential scales.

### 4.3. Results

Manipulation Checks. An independent-samples t-test revealed a significant difference between conditions: participants in the AI condition reported significantly lower scores (*M_AI_* = 2.93, *SD*_AI_ = 0.80) than those in the human condition (*M_human_* = 4. 47, *SD_human_* = 1.17, *t*(228) = 11.71, *p* < 0.001), confirming successful manipulation of the influencer type. For dance style, participants assigned to the modern-style condition (*M_modern-style_* = 4.61, *SD_modern-style_* = 1.18) reported significantly higher agreement than those in the traditional-style condition (*M_traditional-style_* = 3.05, *SD_traditional-style_* = 0.83, *t*(228) = 11.56, *p* < 0.001). These results validate the success of both manipulations.

Main Effects. The main effect of influencer type on purchase intention was not statistically significant (F(1, 226) = 0.89, *p* = 0.35). Similarly, the main effect of dance style was also non-significant (F(1, 226) = 0.47, *p* = 0.50). These findings suggest that neither influencer type nor dance style alone was sufficient to significantly shift participants’ purchase intentions.

Interaction Effects. Importantly, a significant interaction effect was observed between influencer type and dance style (F(1, 226) = 41.67, *p* < 0.001, partial *η*^2^ = 0.161), providing support for H1. This result indicates that the effectiveness of the influencer in promoting food products depends on the style of dance they perform.

**Figure 3:**
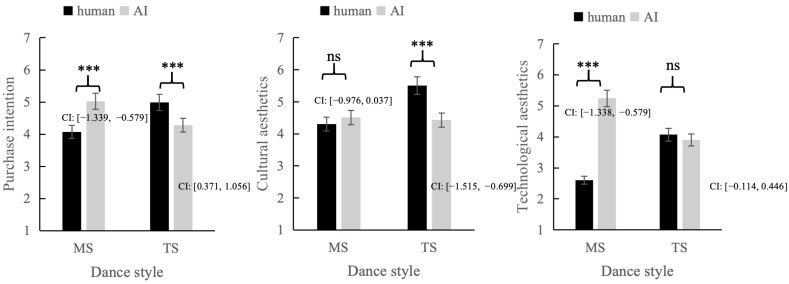
The interaction effects of the influencer type and dance style on purchase intention, cultural aesthetics, and technological aesthetics (Study 2). The error bars show the standard errors of the means. Note. MS: modern-style; TS: traditional-style. That *** denotes *p* < 0.001, ns denotes not significant; CI: confidence intervals.

Simple Effects Analysis. As shown in Figure 3, for adoption intention, the interaction was significant (F(1, 226) = 44.45, *p* < 0.001, partial *η*^2^ = 0.163). Among modern-style condition participants, those exposed to an AI influencer reported significantly greater purchase intentions (*M_AI_* = 5.03, *SD_AI_* = 1.02) than did those exposed to a human influencer (*M_human_* = 4.07, *SD_human_* = 1.06, *t*(116) = −5.01, *p* < 0.001). Among traditional-style condition participants, the pattern reversed: human influencer led to greater purchase intentions (*M_human_* = 4.99, *SD_human_* = 0.81) than AI influencer (*M_AI_* = 4.28, *SD_AI_* = 1.01, *t*(110) = 4.12, *p* < 0.001).

For cultural aesthetics, the interaction was significant (F(1, 226) = 124.71, *p* < 0.001, partial *η*^2^ = 0.51). Among traditional-style condition participants, the human influencer increased cultural aesthetics (*M_human_* = 5.51, *SD_human_* = 0.87) relative to the AI influencer (*M_AI_* = 4.43, *SD_AI_* = 0.53, *t*(121) = −4.94, *p* < 0.001). No significant difference was observed under the modern-style condition (*p* = 0.06).

For technological aesthetics, a similar interaction emerged (F(1, 226) = 9.71, *p* < 0.05, partial *η*^2^ = 0.23). In the modern-style condition, AI influencers led to greater technological aesthetics (*M_AI_* = 5.24, *SD_AI_* = 1.21) than human influencers (*M_human_* = 2.60, *SD_human_* = 1.01, *t*(116) = −12.89, *p* < 0.001). Under traditional-style conditions, there was no significant difference (*p* = 0.24).

Moderated Mediation Analysis. To examine the entire conceptual framework, a moderated dual-mediation analysis was conducted using PROCESS Model 7 (5000 bootstrap samples) [55]. In this analysis, influencer type (AI vs. human) was treated as the independent variable (IV), and dance style (modern vs. traditional) was used as the moderator. Dance style was dummy-coded (0 = modern-style, 1 = traditional-style), and cultural aesthetics and technological aesthetics were the parallel mediators. The indirect effect of the influencer type on purchase intention mediated by technological aesthetics was statistically significant in the modern-style condition (indirect effect = 0.51, 95% CI [0.104, 0.954]). The indirect effect of the influencer type on purchase intention mediated by cultural aesthetics was statistically significant in the traditional-style condition (indirect effect = 0.19, 95% CI [0.071, 0.350]). The other indirect paths were insignificant (all 95% CIs contained zero), which established the fact that moderated mediation did not violate hypotheses H2 and H3.

Behavioural Outcomes. Chi-square analysis showed that participants in the congruent conditions (human–traditional, AI–modern) were more likely to purchase the mandarin (*χ*^2^ = 4.76, *p* < 0.05). This behavioural outcome was consistent with self-reported purchase intentions, reinforcing the ecological validity of the model.

Measurement Model and Discriminant Validity. To validate the measurement model for the aesthetic constructs and alternative mediators, we conducted confirmatory factor analysis (CFA). The CFA results showed that the model fit the data well (CFI = 0.96, TLI = 0.95, RMSEA = 0.04, SRMR = 0.03). We also assessed discriminant validity using the Average Variance Extracted (AVE) and Composite Reliability (CR). The AVE values for cultural aesthetics and technological aesthetics were 0.72 and 0.75, respectively, indicating that each construct explains more than half of its variance. The CR values for the same constructs were 0.92 and 0.93, confirming that the constructs had high internal consistency. Table 1 showing the relationships between the aesthetic constructs and alternative mediators (e.g., trustworthiness and authenticity), is presented below. This matrix shows that all constructs exhibit good discriminant validity, as the AVE for each construct is higher than the squared correlations between them.

Alternative Explanation Tests. In order to exclude the possibility that the dual mediation effects observed can be accounted for by other psychological processes, different alternative mediators, such as perceived trustworthiness, perceived authenticity, and attitude toward the influencer/product, were examined with a series of parallel mediation models through PROCESS Model 7 [55]. All variables were introduced as possible mediators. In particular, the indirect effects through perceived trustworthiness (indirect effect = 0.03, SE = 0.04, 95% CI [ −0. 05, 0.12]), perceived authenticity (indirect effect = 0.02, SE = 0.05, 95% CI [ −0.07, 0.11]), and attitude toward the influencer/product (indirect effect = 0.04, SE = 0.03, 95% CI [ −0.02, 0.09]) were all nonsignificant because the confidence intervals included zero. It is important to note that in a holistic model where these mediators were entered together with cultural aesthetics and technological aesthetics simultaneously, the indirect effects via cultural aesthetics (indirect effect = 0.19, SE = 0.07, 95% CI [0.07, 0.35]) and technological aesthetics (indirect effect = 0.51, SE = 0.21, 95% CI [0.10, 0.95]) still existed and were statistically significant and in the expected directions. These findings preclude other explanations relying on general attitudes or relationships and establish that aesthetic experience is the main process through which influencer–performance congruence is related to consumer purchase intention.

### 4.4. Discussion

The dual aesthetic mediation model has been confirmed in Study 2, as it is a strong support for the fact that the aesthetic experience and not the influencer identity alone can drive consumer reactions to online agrifood marketing. Traditional dancers who are human influencers improved cultural aesthetics [21,22,32], whereas modern dancers who are AI influencers increased technological aesthetics [23,24,33], both causing higher purchase intentions. These results were even more supported by the behavioural choice task, which demonstrated that aesthetic congruence not only determines attitudes but also manifests itself in actual purchasing behaviour.

Furthermore, it was tested that the alternative explanation did not involve other competing psychological processes, such as the perception of trustworthiness, authenticity, and attitude, since no one had a significant indirect effect. Even after controlling those variables, the cultural and technological aesthetic mediation effects still remained significant, indicating that aesthetic congruence is the main channel through which influencer-performance match is related to consumer choice.

## 5. General Discussion

This paper examines how influencer type (AI or human) and dance type (modern or traditional) influence consumer responses in the agrifood marketing context across two experimental studies. The results consistently show that aesthetic congruence, where the visual and symbolic attributes of a performance match the identity of the performer, is linked to increased purchase intentions and actual buying behaviour. Specifically, human influencers dancing traditionally and AI influencers dancing modernly generated the highest levels of consumer responses. These findings suggest that consumers are more likely to be influenced when the sensory elements of the performance align with the meanings they associate with the performer, providing a consistent and engaging aesthetic experience.

Study 2 further explores the underlying mechanisms driving these effects. The results indicate that cultural aesthetics and technological aesthetics serve as two distinct experiential pathways that connect the influencer-performance pairs to consumer purchase behaviour. Human-traditional pairings enhanced feelings of cultural warmth, familiarity, and authenticity, which subsequently led to higher purchase intentions through the cultural aesthetic experience. Conversely, AI-modern pairings evoked novelty, accuracy, and cyber-sophistication, driving the technological aesthetic experience, which also contributed to increased purchase behaviour. Additional analyses were conducted to rule out alternative explanations, such as trustworthiness, authenticity, attitude, and novelty. These analyses confirmed that it was the aesthetic experience itself, rather than general attitude reactions, that directly influenced consumer choices. This finding underscores the importance of the sensory and symbolic aspects of influencer performances, suggesting that consumers are more motivated by the overall aesthetic experience than by individual, cognitive assessments of the influencer’s trustworthiness or authenticity.

### 5.1. Theoretical Implications

This paper contributes to the theoretical knowledge of sensory marketing, aesthetic psychology, and digital influencer literature by introducing a new viewpoint on how performative aesthetics combine with the source identity to affect consumer behaviour. It first adds to the area of sensory marketing by expanding the range of visual cues to include dynamic and embodied cues, namely dance, as an effective means of persuasion in promoting food. Although previous studies have focused more on static sensory components like packaging or colour [5,56], the current paper shows that choreographed movement, which is also a form of performed aesthetics, can evoke emotions and symbolic reactions that are meaningful to purchase decisions.

Secondly, the paper introduces and confirms a dual-pathway model of aesthetic mediation based on the distinction between cultural aesthetics and technological aesthetics. The available aesthetic theories frequently consider consumer reaction to be an overall emotional or perceptual consequence [57,58,59]. Nevertheless, we have found that consumers perceive marketing aesthetics through different symbolic perspectives: cultural aesthetics create a sense of familiarity and authenticity, whereas technological aesthetics signify novelty and precision. These alternative pathways present a deeper insight into the way visual cues in digital environments are handled and integrated, especially when combined with social identity signs, including human or AI representation.

Finally, the study reinterprets the position of AI influencers in the context of marketing communication. Instead of perceiving AI as essentially devoid of authenticity [10,11], the findings indicate that its utility will be determined by aesthetic congruence, i.e., the correspondence between the perceived nature of the influencer and the expressive format of the performance. In combination with current, digitally stylized dance movements, AI influencers may create persuasive effects not by imitating human characteristics but by enhancing their own technological aesthetic. The new understanding provides a new set of theoretical perspectives on symbolic fit, digital persona design, and the development of non-human agents within the consumer culture.

### 5.2. Practical Implications

The results of this study offer several practical implications for marketers, particularly those in agrifood promotion within online settings. First, our findings suggest that aligning the type of influencer with an aesthetic expression that matches their identity can significantly enhance consumer purchase intentions. For brands associated with agriculture or natural foods, using human influencers to perform traditional cultural activities can strengthen the perception of authenticity and locality—factors that are highly influential in consumer assessments of food quality and origin. This finding underscores the continued relevance of traditional aesthetics, which can be effectively leveraged in modern marketing strategies, especially when combined with the human embodiment that consumers still connect with authenticity.

Second, our research highlights that brands targeting younger, digitally savvy audiences on platforms like TikTok or Instagram Reels could benefit from using AI-generated influencers. Instead of replicating human actions, these virtual agents can be presented as futuristic and innovative, especially when paired with modern dance or visually engaging choreography. Such aesthetic congruence not only boosts the perception of innovation but also immerses consumers in the technological aesthetic, offering a differentiated branding approach that takes full advantage of digital novelty without undermining consumer confidence.

Finally, the inclusion of a real behavioural measure in Study 2 reinforces that visual-aesthetic strategies influence more than just attitudes—they also affect actual buying behaviour. For advertisers promoting fresh produce or seasonal food products, incorporating short-form, dance-inspired videos could prove effective in resonating with consumers on an emotional and aesthetic level. These videos should be tailored to fit either a cultural or technological aesthetic schema, as our findings indicate a sensory and symbolic alignment between visual style and source identity. This not only resonates emotionally with consumers but also influences purchase decisions during the buying process.

### 5.3. Limitations and Future Research

This study has several limitations that should be acknowledged. First, although the manipulation checks were statistically significant, we acknowledge that the strength of the manipulation was moderate. Future research could refine manipulation checks to ensure that differences are strictly due to influencer type and dance style manipulations. Additionally, the use of a single cultural context (Chinese university students) and a narrow product category (fresh food products) limits the generalizability of our findings. The associations with tradition and naturalness in food products may not apply to processed or technology-driven food categories. Future studies should explore a broader range of product categories, such as packaged foods, plant-based alternatives, or functional foods. Moreover, while we tested trustworthiness, authenticity, and attitude as mediators, these constructs may be downstream of aesthetic experiences. Testing them in isolation does not fully rule out the possibility of sequential mediation, where aesthetics influence authenticity, which in turn affects purchase intention. Future research could explore serial mediation models to better understand these complex pathways. Finally, the cultural context of this study was limited to Chinese participants, and the concept of traditional dance may not carry the same meaning in other cultures. Future research should examine how these aesthetic congruence effects apply in different cultural contexts [60].

## Figures and Tables

**Table 1:** Correlation matrix.

	CA	TA	TW	AU	AT
CA	1				
TA	0.45	1			
TW	0.35	0.33	1		
AU	0.48	0.5	0.4	1	
AT	0.42	0.37	0.55	0.6	1

## Data Availability

The original contributions presented in the study are included in the article, further inquiries can be directed to the corresponding author.
